# Factors affecting the daily feed intake and feed conversion ratio of pigs in grow-finishing units: the case of a company

**DOI:** 10.1186/s40813-016-0023-4

**Published:** 2016-03-01

**Authors:** C. R. Pierozan, P. S. Agostini, J. Gasa, A. K. Novais, C. P. Dias, R. S. K. Santos, M. Pereira Jr, J. G. Nagi, J. B. Alves, C. A. Silva

**Affiliations:** 1 Departamento de Zootecnia, Universidade Estadual de Londrina, 86051-970 Londrina, Brazil; 2 Grup de Nutrició, Maneig i Benestar Animal, Department de Ciència Animal i dels Aliments, Universitat Autònoma de Barcelona, 08193 Bellaterra, Spain

**Keywords:** Feed intake, Feed conversion ratio, Grow-finishing pigs, Production factors

## Abstract

**Background:**

The aim of this study was to use mathematical modeling to identify and quantify the main factors that affect daily feed intake (DFI) and feed conversion ratio (FCR) in grow-finishing (GF) pig units. We evaluated the production records of 93 GF farms between 2010 and 2013, linked to a company, working in a cooperative system, located in western Paraná State, Brazil. A total of 683 batches, consisting of approximately 495,000 animals, were used. Forty production factors related to the management, health, plant and equipment, nutrition, genetics and environment were considered. The number of pigs per pen, type of feeder, origin and sex (the last two variables were combined in the models) of the animals and initial and final body weights were included in the final models to predict DFI and FCR (dependent variables). Additionally, the duration of the GF phase was included for the parameter FCR. All factors included in the final models had significant effects for both dependent variables.

**Results:**

There was a reduction in DFI (0.04 kg) (P < 0.001) and an improvement in FCR (6.0 points) (P < 0.001) in batches from pens with less than 20 animals compared with batches from pens with more than 20 animals. In barns with “other” feeder types (mostly the linear dump type) different of conical semiautomatic feeder, a reduction of DFI (0.03 kg) (P < 0.05) and improved FCR (3.0 points) (P < 0.05) were observed. Batches of barrows from units specialized for producing piglets (SPU) had higher DFI (approximately 0.02 kg) (P < 0.01) than batches of females and batches of mixed animals from SPU, and batches of mixed animals from farms not specialized for piglet production (farrow-to-finish farms). Batches of females from SPU and mixed batches from SPU had better FCR (5.0 and 3.0 points respectively) (P < 0.001 and P < 0.001, respectively) than batches of piglets originating from farrow-to-finish farms. The variables selected for the final models explained approximately 50 and 64 % of the total variance in DFI and FCR, respectively.

**Conclusions:**

The models are tools for the interpretation of the factors related to the evaluated parameters, aiding in the identification of critical aspects of production. The main parameters affecting DFI and FCR in this company during the GF period were the number of pigs per pen, the type of feeder used and the combination origin-sex of the animals.

## Background

Feed accounts by approximately 65–75 % of pig production cost and 75 % of that feed consumed in the grow-finishing (GF) phase [1]. Despite the economic importance of the GF phase, few studies of Brazilian farms have aimed to quantify the effect of the main production factors over the performance of GF pigs. Although the major factors affecting pig performance are known [2–4], such as genetics, nutrition and feeding, housing conditions and health, studies relating these variables with each other, especially genetics to nutrition and feeding [5, 6] and health [7] are scarce. Those that relate production parameters to the conditions of facilities and equipment involved [8] are even scarcer. Agostini et al. [9] have established a relationship among production factors and performance indexes from more than one million pigs in GF phase from eight different companies in Spain. From the results important recommendations were made, both for immediate changes in feeding, nutrition and management and for future action in genetics, construction and environmental issues. The same authors [10] also indicated that models within company are more reliable than models obtained among companies, since each company has its specific management, nutrition and facilities features across its farms.

When evaluating the effects of production factors upon a specific livestock parameter, mathematical models are a potentially effective tool. These models are primarily intended to represent a simplification of reality that from a mathematical point of view, describes a phenomenon based on factors of interest [11]. The use of modeling has allowed researchers in agricultural systems to develop concepts, methods and tools to direct the activity as a whole [12]. According to Dent et al. [13], the model construction process itself contributes to a better understanding and description of a given system.

The aim of this study was to use mathematical models to identify and quantify the impact of various intrinsic and extrinsic production factors on the daily feed intake (DFI) and the feed conversion ratio (FCR) in grow-finishing (GF) pig farms of a single company. The results may help company managers to predict the production rates and to focus their limited resources in the areas of higher profit.

## Methods

### Data collection

Animal Care and Use Committee approval was not necessary as this study used a database of a survey carried out in existing commercial farms.

Between 2010 and 2013, the historical production parameters of 683 batches of pigs in GF phase (totaling approximately 495,000 animals) from all the 93 farms (7.34 batches per farm) integrated to a company located in Western Paraná (Brazil) were used.

The workflow followed the study conducted by Agostini et al. [9] and was developed in two stages. In the first stage the variables of interest were chosen, representing the most important factors affecting the livestock production records of the company. Then a model that offers reliability, speed and efficiency in collecting the information was later established. Differently from the study of Agostini et al. [9], the data belong to all farms integrated in the company and with a greater number of batches per farm. All farms provided batches from different seasons.

The dependent and independent variables were selected by taking into account recent scientific work and the field experience of the company’s staff. The dependent variables choose were the DFI and FCR. The total feed intake per animal was calculated as the total amount of feed in kilograms delivered to each batch during the GF period, minus the amount of feed remained in the silos when the animals were sent to slaughter, divided by the number of pigs marketed. Then the DFI was calculated as the total feed intake per animal divided by the average number of days that the animals remained in the GF unit. FCR was obtained by dividing the total feed intake of each batch by the difference between the total kilograms of pigs sent to slaughter and the total kilograms of pigs that entered at the GF batch. Mortality rate was not considered in the calculations of DFI and FCR since feed intake and body weight of dead animals were not registered.

Initially, four continuous independent variables were evaluated: number of pigs placed (NPP), initial weight (IW), final weight (FW), and duration of GF phase (DGF) as presented in Table 1. The NPP was the total number of pigs housed in the GF units. The IW corresponded to the pigs’ live weight in kilograms when they entered the GF units, and the FW to the average live weight of pigs at slaughter. The DGF was the period, in days, that animals remained in the GF unit. Because the data concerning the NPP were not normally distributed, this variable was considered as categorical.

**Table 1:** Descriptive values of dependent and independent continuous variables selected for the final models

Variable	N° batches	Mean	SD	Minimum	1^st^ quartile	Median	3^rd^ quartile	Maximum
Number of pigs	683	726	430	200	499	608	919	2393
IW (kg)	683	22.7	1.2	18.9	22.2	22.8	23.4	27.6
FW (kg)	683	117	5	100	113	117	120	132
DGF (day)	683	107	4	96	104	107	110	120
DFI (kg/pig)	682	2.15	0.10	1.82	2.09	2.15	2.22	2.48
FCR (kg/kg)	682	2.45	0.12	2.15	2.36	2.45	2.54	2.86

*SD* standard deviation, *IW* initial weight, *FW* final weight; *DGF* duration of growing-finishing phase, *DFI* daily feed intake, *FCR* feed conversion ratio

Approximately forty categorical independent variables were also evaluated (Table 2) that represented factors of production related to facilities, herd health, and aspects of livestock management systems and nutrition. To obtain this information, questionnaires were given both as digital spreadsheets (Excel 12.0, Office 2007) and on paper.

**Table 2:** Description of independent categorical variables and their percentage of occurrence in the company

Variable	Percentage of batches in each category
Semester of placement^b,e^	Summer / autumn (48.76 %); winter / spring (51.24 %)
Number of animals placed^b,f^	< 500 (20.78 %); 500–1000 (55.04 %); > 1000 (24.18 %)
Number of barns^b,f^	One (42.14 %); two or more (57.86 %)
Stall age^b,f^	< 5 years (20.78 %); 5 to 10 years (53.26 %); > 10 years (25.96 %)
Reform of facilities^b,f^	Yes (21.07 %); no (78.93 %)
Number of pigs per pen ^b,c,f^	< 20 (21.81 %); > 20 (78.19 %)
Building material/ barn^a,f^	Masonry (97.48 %); wood and mixed (2.52 %)
Type of feeder^b,c,f^	Conical semiautomatic (81.75 %); others (18.25 %)^d^
Type of drinker^a,f^	Nipple (98.66 %); water cup (1.34 %)
Water source^b,f^	Well / headwater (55.19 %); treated water (44.81 %)
Water pipes material^a,f^	Hose (1.48 %); PVC pipe (97.18 %); mixed (1.34 %)
Roof material^b,f^	Clay (87.39 %); asbestos / zinc (12.61 %)
Material used to separate the pens^b,f^	Wood or masonry (18.69 %); mixed (81.31 %)
Floor material^a,f^	Concrete (100 %)
Pens with shallow pools^a,f^	Yes (99.85 %); no (0.15 %)
Slurry tank^a,f^	Yes (100 %)
Electricity supply^a,f^	Yes (100 %)
Waste lagoons^a,f^	Yes (100 %)
Ventilation fans^a,f^	Yes (2.52 %); no (97.48 %)
Exhaust fans^a,f^	No (100 %)
Humidifiers / nebulizers^b,g^	Yes (25.71 %); no (74.29 %)
Composters^a,f^	Yes (98.37 %); no (1.63 %)
Trees around the facilities^b,f^	Yes (43.62 %); no (56.38 %)
Barn’s position relative to the sun^b,f^	Diagonal / contrary (44.07 %); parallel (55.93 %)
Number of feed used^a,f^	Five (100 %)
Different feeds according to the sex^a,f^	No (100 %)
Feed form^a,f^	Pelleted (100 %)
Shock with antibiotics^a,f^	Yes (100 %)
Routes used to administer antibiotics^a,f^	Water (1.19 %); water and feed (98.81 %)
Programs used^a,f^	Ractopamine / immunocastration (100 %)
Labour force^b,f^	Unfamiliar (24.48 %); familiar (75.52 %)
Number of employed genetic^a,f^	Three (100 %)
Breeds used^a,e^	Large White / Landrace / Pietrain (100 %)
Sexed batches^a,f^	No (100 %)
Sex segregation in pens^a,f^	Yes (100 %)
Ileitis, enzootic pneumonia, meningitis^a,e^	Yes (100 %)
Glasser’s disease, erysipela^a,e^	No (100 %)
Origin^b,c,e,i^	SPU (42.9 %); farrow-to-finish units (57.1 %)
Sex^b,c,h^	Barrows (11.85 %); females (12.92 %); mixed (75.23 %)

^a^Variables initially rejected to the statistical analysis due to the absence of variability among its categories

^b^Variables initially considered to the statistical analysis

^c^Variables included in the final models

^d^Others: composed mostly by linear dump type (17.2 %) and a few farms with a linear semiautomatic one (1.1 %)

^e^Considering 683 batches as experimental units (n)

^f^ *n* = 674

^g^ *n* = 669

^h^ *n* = 650

^i^Percentage of batches composed by animals coming either from a specialized piglet production unit (SPU) or from different farrow-to-finish units

### Statistical analysis

The collected data were entered into an Excel spreadsheet before statistical analysis was carried out. The analysis was done in two phases: exploratory analysis and model development as previously carried out by Oliveira et al. [8], Agostini et al. [14] and Maes et al. [15]. In the exploratory analysis phase, a frequency study of the categorical variables was conducted using the SAS FREQ procedure (SAS Inst., Inc., Cary, NC, USA, version 9.2) (occurrence percentages in Table 2). Categorical variables with absence of variability among their categories (more than 90 % of the total batches included to a given category) were initially excluded for further statistical analysis (Table 2).

Measures of central tendency (mean and median) and dispersion (standard deviation, quartiles and amplitude) for the continuous variables were computed using the SAS MEANS procedure (Table 1). The distributions of continuous variables were evaluated using the SAS UNIVARIATE procedure. In all these analyses, the batch was considered the experimental unit, defined as a single group of piglets that came from the nursery phase and were housed in a GF unit until slaughter. All batches were managed as all-in all-out systems.

Mixed linear regression models were fit using the SAS MIXED procedure, using the variables that were coded in the first phase as predictors. The effect of farm and batch within the farm were considered as random factors, and the variance was estimated using the restricted maximum likelihood method. The comparison of the final models’ goodness of fit was based on the proportion of variance explained by the different models, using the coefficient of determination (R^2^) as a parameter.

In the second phase, a single regression model was used where each variable was included as a fixed effect for each single dependent variable. The independent variables with P ≤ 0.20 were selected for use in the multivariate analysis.

Pearson and Spearman correlations were performed between independent variables to avoid multicollinearity between continuous variables and confounding problems between categorical variables. When two variables had high correlation coefficients (absolute value ≥ 0.60), only one was used in the multivariate analysis; the choice between them was made by comparing the *P* values in the univariate analysis, and additionally evaluating their biological relevance with respect to the dependent variable. In that case the variables “origin” and “sex” of the animals showed a relationship being used only in particular combinations and hence both were grouped as a single combined variable (ORIGSEX).

Subsequently, all independent variables selected in the univariate analysis were submitted to the procedure “stepwise”, where all factors with *P* < 0.05 were kept in the final multivariate model. Fixed-effect testing was based on the F-test with denominator degrees of freedom approximated by the Satterthwaite’s procedure. Significant interactions (*P* < 0.05) between the variables in the multivariate model were tested and included.

After obtaining the models for each dependent variable, the residuals were plotted against the predicted values to check the homogeneity of variances and the presence of outliers. All the factors with *P* < 0.05 in the final models for each of the two dependent variables (DFI and FCR) were considered statistically significant.

## Results

### Daily feed intake

**Fig. 1:**
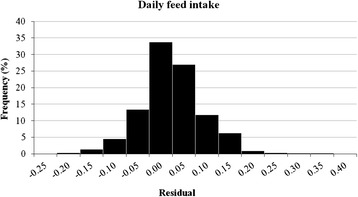
Residual distribution of the effects of the factors studied on daily feed intake in 683 batches from 93 grow-finishing pig farms

The DFI per pig per batch was 2.15 ± 0.10 kg (ranging from 1.82 to 2.48 kg) (Table 1). Multivariate regression analysis indicated that DFI was influenced by the number of pigs per pen (*P* < 0.001), type of feeder (*P* = 0.03), ORIGSEX (*P* = 0.01), IW (*P* < 0.001) and FW (*P* < 0.001) (Table 3). The total variance of DFI in the model without predictors (the null model) was 0.009541, where 0.00346 (36.3 %) was observed between farms and 0.006081 (63.7 %) between batches from the same farm. After the variables were included in the multivariate model, the residual variance for the DFI was reduced to 0.004806, which indicated that approximately 50 % of the total variance of DFI was explained by the variables included in the final model (Table 4). The residual distribution of DFI is highlighted in Fig. 1. The percentages of the variance explained between farms and between batches within a farm, using the final model for DFI, were 60.8 and 43.3 %, respectively (Table 4).

**Table 3:** Estimates of the effects of the factors studied on daily feed intake (in kilograms per pig) in 683 batches from 93 grow-finishing pig farms

Variable	Category	Mean (kg)	Estimate (s.e.)	95 % CL		
				Low	Upper	*P*-value
Intercept		−	0.73 (0.07)	0.58	0.88	< 0.001
N^o^ pigs per pen	< 20	2.11	−0.04 (0.01)	−0.06	−0.02	< 0.001
	> 20	2.16	0	–	–	–
Type of feeder	Others (linear dump)	2.12	−0.03 (0.01)	−0.05	−0.003	0.03
	Conical semiautomatic	2.15	0	–	–	–
ORIGSEX	SPU / barrows	2.15	0.02 (0.01)	0.005	0.04	0.009
	SPU / females	2.12	−0.01 (0.01)	−0.03	0.003	0.12
	SPU / mixed	2.13	0.0004 (0.0071)	−0.013	0.014	0.95
	Farrow-to-finish / mixed	2.13	0	–	–	–
IW		–	0.008 (0.002)	0.004	0.013	< 0.001
FW		–	0.01 (0.00)	0.01	0.01	< 0.001

*s.e.* standard error, *CL* confidence level, *ORIGSEX* variables ¨origin ¨ and ¨sex¨ combined, *SPU* specialized piglet production unit, *IW* initial weight, *FW* final weight

**Table 4:** Variance observed between farms and between batches within a farm for model without predictors (null model) and multivariate model (full model) and percentage of variance explained by the variables included in the final model for daily feed intake

	Null model		Full model		Variance explained (%)
Effect	Variance	%	Variance	%	
Farm	0.00346	36.3	0.00136	28.2	60.8
Batches (Farm)	0.00608	63.7	0.00345	71.8	43.3
Total	0.00954	100.0	0.00481	100.0	49.6

In batches with less than 20 animals per pen, the DFI per pig was lower (0.04 ± 0.01 kg) than in batches with more than 20 animals. In pens where the feeder was not semiautomatic (of these, the most common type was the linear dump one), a reduction of DFI was observed (approximately 0.03 ± 0.01 kg). A higher DFI (approximately 0.02 ± 0.01 kg) was found in batches of barrows from SPU than in batches of females from SPU and batches of animals of mixed sex from both SPU and farrow-to-finish farms. The regression analysis indicated that for each kilogram of IW, there was an increase of approximately 0.008 ± 0.002 kg in DFI, and for each kilogram of FW, DFI increased by approximately 0.01 ± 0.0005 kg, as presented in Table 3.

### Feed conversion ratio

**Fig. 2:**
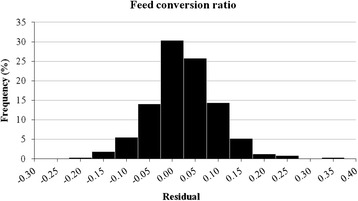
Residual distribution of the effects of the factors studied on feed conversion ratio in 683 batches from 93 grow-finishing pig farms

The average FCR was 2.45 ± 0.12 (range 2.15 to 2.86) (Table 1). Multivariate regression analysis showed that FCR was influenced by the number of pigs per pen (*P* < 0.001), type of feeder (*P* = 0.04), ORIGSEX (*P* < 0.001), IW (*P* < 0.001), FW (*P* < 0.001) and DGF (*P* < 0.001) (Table 5). The model without predictors (the null model) for FCR had a total variance of 0.015261, where 0.002331 (15.3 %) was observed between farms whereas 0.01293 (84.7 %) was between batches from the same farm. The multivariate model reduced the residual variance of FCR to 0.005516, which indicated that approximately 64 % of its total variance was explained by the predictors in the final model (Table 6). The residual distribution of FCR is shown in Fig. 2. The percentages of variability explained between farms and between batches from the same farm were 33.5 and 69.3 %, respectively (Table 6).

**Table 5:** Estimates of the effects of the factors studied on feed conversion ratio in 683 batches from 93 grow-finishing pig farms

Variable	Category	Mean (kg/kg)	Estimate (s.e.)	95 % CL		
				Low	Upper	*P*-value
Intercept		–	1.43 (0.10)	1.23	1.62	< 0.001
N^o^ pigs per pen	< 20	2.40	−0.05 (0.01)	−0.07	−0.03	< 0.001
	> 20	2.45	0	–	–	–
Type of feeder	Others (linear dump)	2.41	−0.03 (0.01)	−0.06	−0.00	0.04
	Conical semiautomatic	2.44	0	–	–	–
ORIGSEX	SPU/barrows	2.43	−0.02 (0.01)	−0.03	0.00	0.09
	SPU/females	2.40	−0.05 (0.01)	−0.07	−0.03	< 0.001
	SPU/mixed	2.42	−0.03 (0.01)	−0.04	−0.01	< 0.001
	Farrow-to-finish/mixed	2.44	0	–	–	–
IW		–	0.035 (0.002)	0.03	0.04	< 0.001
FW		–	−0.01 (0.00)	−0.01	−0.01	< 0.001
DGF		–	0.015 (0.001)	0.01	0.02	< 0.001

*s.e.* standard error, *CL* confidence level, *ORIGSEX* variables ¨origin ¨ and ¨sex¨ combined, *SPU* specialized piglet production unit, *IW* initial weight, *FW* final weight, *DGF* duration of the grow-finishing phase

**Table 6:** Variance observed between farms and between batches within a farm for model without predictors (null model) and multivariate model (full model) and percentage of variance explained by the variables included in the final model for feed conversion ratio

	Null model		Full model		Variance explained (%)
Effect	Variance	%	Variance	%	
Farm	0.00233	15.3	0.00155	28.1	33.5
Batches (Farm)	0.01293	84.7	0.00397	71.9	69.3
Total	0.01526	100.0	0.00552	100.0	63.9

Feed conversion ratio improved by 6.0 ± 1.2 points when animals were kept at less than 20 per pen compared to batches with more than 20 animals per pen. The type of feeder had also an effect, with non-automatic feeders (mainly linear dump type) improving FCR by 3.0 ± 1.4 points. Regarding ORIGSEX, there was an improvement in FCR of approximately 5.0 ± 1.0 points for batches of females from SPU and 3.0 ± 1.0 points for mixed-sex batches from SPU compared to mixed-sex batches from farrow-to-finish farms. Multivariate regression analysis showed that FCR improved by approximately 3.5 ± 0.2 points for each additional kilogram of IW and by approximately 1.0 ± 0.0 points for each additional kilogram of FW. The DGF also influenced FCR, with each day in the GF phase being approximately 1.5 ± 0.0 points worse, as demonstrated in Table 5.

## Discussion

In this study, all the factors included in the final models had an influence on the dependent variables, and the total variance of DFI and FCR accounted by the models was 50 and 64 %, respectively. The final models developed by Agostini et al. [14] explained 62 % of the total variance of total feed intake and 24.8 % of FCR whereas the one developed by Oliveira et al. [8] explained 81 % of the total variance of DFI. The difference between the percentages of variance explained in these studies may be due to the difference between the variability of the factors studied.

One aspect observed in this study concerns the variability explained between farms and between batches within a farm with the multivariate models of DFI and FCR. Approximately 43.3 and 69.3 % of the variability of DFI and FCR, respectively, was explained between batches from the same farm. This greater proportion of the variability between batches explained in a farm for FCR is due to the inclusion of the variable DGF (a variable taken per batch, and not per farm) in the model; this variable is not included in the model for DFI.

With respect to the number of pigs per pen, there was a decrease of DFI and improve of FCR in pens that had less than 20 animals throughout the GF phase. The analysis of the social changes due to an increased number of animals in the pen has a great importance for animal welfare and for productivity [16]. According to Schmolke et al. [17], one concern about large group size is the reduced growth rate. Street and Gonyou [18] found that pigs housed in small groups (18 animals) during the GF phase reached 3 % more weight than those housed in large ones (108 animals). FCR was also better (6 %) in small groups, and this was more evident at the end of the study (14 % more efficient than those housed in large groups). These results were similar to those found by Vermeer et al. [19], who found that pigs kept in larger groups in the GF phase grew slower. A large group size can provide greater opportunities for exploration and freedom of movement. Thus, during the grower phase, the poorer growth rate may be explained by the fact that part of dietary energy is rather directed to satisfy the demands of greater locomotor activities of animals [20], resulting in worse FCR. Some pig companies may choose to build larger pens (with more animals per pen but no change in space allowance per animal) to better use the space of the barns by reducing the area that would be used to runners and partitions. However, the increase in efficiency offered by the construction of large spaces together with the reduction of work required per pig must be counterposed to the reduction in growth rate during the phases of post-weaning and growth when animals are housed in large groups [20]. Anyway, new studies on the subject should take into account the statements made by Estevez et al. [16], in which the search for the so-called “optimum group” is somewhat guaranteed to fail. Group sizes will vary not only according to the animal species but also according to the complexity of environmental factors involved, such as the availability and location of food.

The type of feeder significantly affected DFI and FCR. The use of “other” feeder types (most commonly, the linear dump one) resulted in reduced DFI and better FCR over the use of conical semiautomatic feeder. The cost and the need to modify the facilities make experiments with different types of feeders difficult to conduct [21]. Studies that have related the performance parameters between two type of feeders have included comparisons between those that simultaneously provide feed and water to feed animals with those that offer only dry feed [14, 22, 23], comparisons between feeders with a single space for animals versus those that offer multiple spaces [14, 22–24], and evaluations of the effects of changing the type of feeder between the growing and finishing phases [25]. Changing feeder when animals are transferred to GF housing leads to reduced feed intake and performance in the first week after the change, but no negative effect on performance over the entire finishing phase [25]. Comparisons with the results of this study are therefore limited, given the limited information on the subject and because of the wide variation in existing feeders already studied.

The type of feeder may influence feed wastage. In commercial farm conditions, as feed wastage is not subtracted from the actual consumption, the increased wastage results in higher DFI and worse FCR, since the feed is not being utilized for animal growth. Agostini et al. [14] observed a reduction in feed intake without affecting weight gain, when pigs were fed in troughs that provided a unique space associated with a drinker. This could be due to the lower feed wastage with this equipment. In the present study the lower DFI observed in pigs fed in non-conical semiautomatic feeders (mainly linear dump one) might be related to the reduced feed wastage, which leads to an enhanced FCR. In this regard, the regulation of feeders should be considered because when these equipment, such as conical semiautomatic feeders, are inappropriately regulated, feed wastage can increase significantly.

Regarding the variables ORIGSEX, barrows from SPU have a higher DFI than females and mixed-sex batches from SPU as well as mixed-sex batches from farrow-to-finish farms. As for FCR, batches of females and mixed-sex batches from SPU showed better performance than batches of barrows originating from SPU and mixed-sex batches from farrow-to-finish farms.

The company evaluated in the present study has one specialized piglet production unit (SPU), which produces about 1,600 piglets per week that are housed in GF units. SPU are very common in the Brazilian pig industry. Commonly they adopt all-in all-out management, ensuring better health for the animals sent to GF farms. However, some GF units also receive piglets from farrow-to-finish farms [26], whose investments in animal health are usually smaller. Therefore, it is not uncommon for some of these farms to also be close to other farms without proper biosecurity, which facilitates the transmission of infectious agents. The better health of piglets from SPU may explain their higher DFI and better FCR.

For sex, these results corroborate those of Morales et al. [27] who observed a higher DFI (6.1 %) for barrows than for females in the GF phase (from 62 to 174 days of age), and Bünzen et al. [28] who observed that males can consume from 10 to 19 % more feed between 60 and 105 kg than females. Sundrum et al. [29] and Brustolini and Fontes [30] showed that due to the lower feed intake, females require approximately six days extra to reach the slaughter weight of 120 kg.

## Conclusions

In the evaluated conditions, the results showed that GF pigs had a higher DFI and worse FCR when: a) housed in pens with more than 20 animals, b) fed in conical semiautomatic feeder and c) batches were composed by barrows coming from specialized piglet production unit and mixed-sex coming from farrow-to-finish units.

The design of this study gives to pig company and their farms a way to predict the weight of these factors on their performance indices and it seems to be an effective tool to assist technicians and producers in taking management decisions.
